# A Functional 12T-Insertion Polymorphism in the *ATP1A1* Promoter Confers Decreased Susceptibility to Hypertension in a Male Sardinian Population

**DOI:** 10.1371/journal.pone.0116724

**Published:** 2015-01-23

**Authors:** Victoria L. Herrera, Khristine A. Pasion, Ann Marie Moran, Roberta Zaninello, Maria Francesca Ortu, Giovanni Fresu, Daniela Antonella Piras, Giuseppe Argiolas, Chiara Troffa, Valeria Glorioso, Wanda Masala, Nicola Glorioso, Nelson Ruiz-Opazo

**Affiliations:** 1 Hypertension and Related Diseases Center, AOU-Universita’ di Sassari, Sassari, Sardinia, Italy; 2 Whitaker Cardiovascular Institute, Department of Medicine, Boston University School of Medicine, Boston, Massachusetts, United States of America; Instituto de Higiene e Medicina Tropical, PORTUGAL

## Abstract

Identification of susceptibility genes for essential hypertension in humans has been a challenge due to its multifactorial pathogenesis complicated by gene-gene and gene-environment interactions, developmental programing and sex specific differences. These concurrent features make identification of causal hypertension susceptibility genes with a single approach difficult, thus requiring multiple lines of evidence involving genetic, biochemical and biological experimentation to establish causal functional mutations. Here we report experimental evidence encompassing genetic, biochemical and *in vivo* modeling that altogether support *ATP1A1* as a hypertension susceptibility gene in males in Sardinia, Italy. *ATP1A1* encodes the α1Na,K-ATPase isoform, the sole sodium pump in vascular endothelial and renal tubular epithelial cells. DNA-sequencing detected a 12-nucleotide long thymidine (12T) insertion(ins)/deletion(del) polymorphism within a poly-T sequence (38T vs 26T) in the *ATP1A1* 5’-regulatory region associated with hypertension in a male Sardinian population. The 12T-insertion allele confers decreased susceptibility to hypertension (P = 0.035; OR = 0.50 [0.28–0.93]) accounting for 12.1 mmHg decrease in systolic BP (P = 0.02) and 6.6 mmHg in diastolic BP (P = 0.046). The *ATP1A1* promoter containing the 12T-insertion exhibited decreased transcriptional activity in in vitro reporter-assay systems, indicating decreased α1Na,K-ATPase expression with the 12T-insertion, compared with the 12T-deletion *ATP1A1* promoter. To test the effects of decreased α1Na,K-ATPase expression on blood pressure, we measured blood pressure by radiotelemetry in three month-old, highly inbred heterozygous knockout *ATP1A1+/−* male mice with resultant 58% reduction in ATP1A1 protein levels. Male *ATP1A1+/−* mice showed significantly lower blood pressure (P < 0.03) than age-matched male wild-type littermate controls. Concordantly, lower ATP1A1 expression is expected to lower Na-reabsorption in the kidney thereby decreasing sodium-associated risk for hypertension and sodium-induced endothelial stiffness and dysfunction. Altogether, data support *ATP1A1* as a hypertension susceptibility gene in a male Sardinian population, and mandate further investigation of its involvement in hypertension in the general population.

## Introduction

Essential hypertension is a complex multifactorial condition influenced by both genetic and environmental factors [1]. As a complex polygenic disorder, identification of hypertension susceptibility genes has been difficult requiring multiple lines of evidence to prove their causal roles in essential hypertension pathogenesis [2,3]. The α1 Na,K-ATPase (ATP1A1) is the sole active Na^+^ transporter in the renal basolateral epithelia throughout the nephron [4–6] and in vascular endothelium [7], and given the role of sodium as a major risk factor for hypertension, the ATPA1 gene is a logical candidate gene for susceptibility to salt-sensitive ‘essential’ or polygenic hypertension [4–6]. The investigation of *ATP1A1* in human essential hypertension is supported by cumulative evidence obtained in the Dahl salt-sensitive hypertensive rat model, linking *ATP1A1* to salt-sensitive hypertension [8–12], documenting a functionally significant variant [8] confirmed by ligase chain reaction-assays [13], protein Edman degradation sequencing and amplification-independent allele-specific PCR-assays [11,13]. Furthermore, rescue of the salt-sensitive hypertensive phenotype was attained through transgenic expression of the ‘wild-type’ Dahl salt-resistant *ATP1A1* variant in Dahl S rats [10].

In humans, *ATP1A1* single-point and haplotype association analyses of a Sardinian hypertensive/normotensive > 60 yrs cohort demonstrated gender-specific association of the *ATP1A1* locus with hypertension in males [14]. These results corroborated earlier reports linking the *ATP1A1* locus with hypertension susceptibility in a Quebec family study [15] and our earlier observations in the same Sardinian cohort using microsatellite markers in close proximity to the *ATP1A1* locus [16]. The association of the haplotype defined by single nucleotide polymorphisms (SNPs) located in the *ATP1A1* 5’ flanking regulatory region suggested that the *ATP1A1* promoter region is a likely location of putative molecular variants contributing to modulation of hypertension susceptibility in this population [14]. Given the association of *ATP1A1* with hypertension in a rat model and in a Sardinian cohort, and the following facts: that ATP1A1 is the sole Na,K-ATPase α-subunit isoform in vascular endothelial cells [7] and renal tubular epithelial cells involved in Na-reabsorption [5,6], that increased sodium levels is implicated in endothelial stiffness [17,18], and that sodium is a known risk factor for arterial stiffness [19] and hypertension [5], it becomes apparent that the *ATP1A1* gene is a logical candidate hypertension gene. We therefore, investigated putative functionally-significant DNA sequence-variants within the *ATP1A1* 5’ regulatory region that might contribute to susceptibility or resistance to essential hypertension in the Sardinian cohort of hypertensive and normotensive people > 60 years of age queried in the association study [14].

## Results

### Scanning of *ATP1A1* 5’-regulatory region for candidate variants

To elucidate potential variants within the *ATP1A1* promoter region we cloned and sequenced a 4551 bp DNA fragment from three patients carrying haplotypes associated with hypertension and from three subjects carrying haplotypes associated with normotension respectively that were previously identified in the Sardinian cohort in prior association studies [14]. We note that normotensive individuals were limited to 60 years and older to ascertain normotension ≥ 60 years, and since hypertensive-normotensive patient age-matching is not important given that age differences do not affect DNA-sequence variants, unlike physiological or biochemical assays.

**Figure 1:**
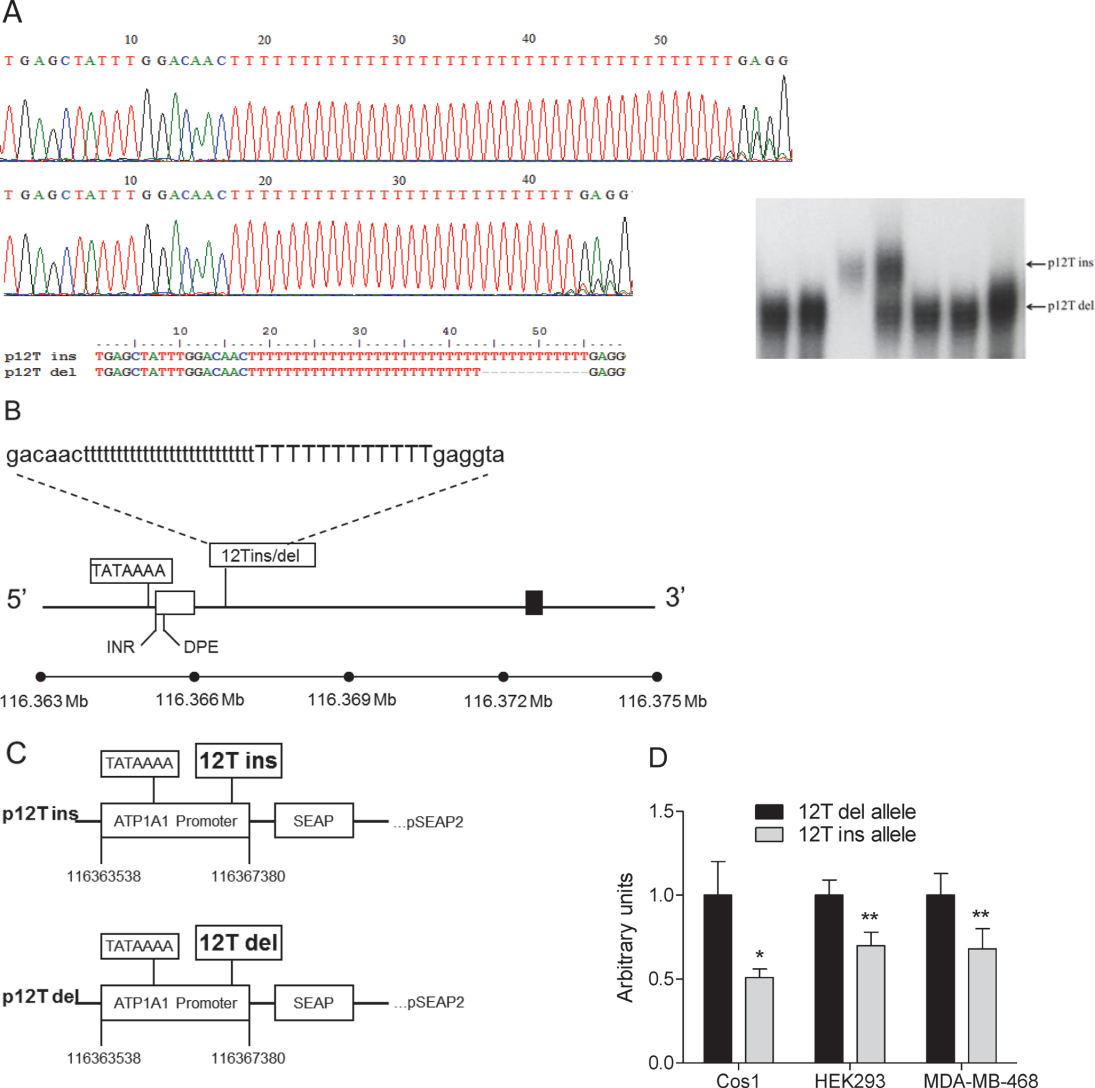
Identification of the 12T-ins/del polymorphism in the *ATP1A1* promoter region and transcriptional activity of *ATP1A1* promoter variants. (A) Nucleotide sequence spanning the poly-T sequence involved in the 12T-ins/del polymorphism from the two *ATP1A1* (p12T ins, p12T del) reporter gene constructs utilized in the transcriptional assays. On right detection of 12T-insertion and 12T-deletion alleles by PCR-amplification followed by denaturing polyacrylamide gel (6%) electrophoresis used for genotyping of the Sardinian cohort. (B) Illustration of the *ATP1A1* promoter region. Non-coding exon is presented as open box and exon encoding the NH_2_-terminal region is presented as black box. Sequence and location of the *ATP1A1* 12T-ins/del polymorphism is shown. The positions of TATAAA box, INR (initiator) and DPE (downstream promoter element) core promoter elements within *ATP1A1* promoter are shown. Core promoter elements were identified based on 100% homology with corresponding consensus sequences [39,40]. (C) Schematic of two *ATP1A1* (p12T ins, p12T del) reporter gene constructs. (D) Relative transcriptional activity of 12T-insertion (p12T ins) and 12T-deletion (p12T del) gene constructs in Cos1, HEK293 and MDA-MB-468 cells. *, *P* < 0.05; **, *P* < 0.01 (two-tailed student *t*-test).

We used as forward primer: 5′-AGA-TCA-TGA-GGC-TGA-GTG-AG-3′ and as reverse primer: 5′-TTC-CAT-TTT-GGC-GAT-GGT-G-3′ which span chromosome (Chr)-1 coordinates 116363538–116368089 of the 5’-regulatory region of *ATP1A1* (Fig. 1). DNA sequence analysis detected a 12T-insertion/deletion (12T-ins/del) polymorphism involving a poly-T sequence (38T/26T) in close proximity to a putative TATAAA-box in the promoter region of the *ATP1A1* gene (Fig. 1A–1B) that could potentially modulate *ATP1A1* transcription. Concordantly, a potential RNA initiation site is identified by consensus sequence in close proximity to nucleotide 116,366,000 within the predicted exon 1 (5’-Untranslated region) and supported by RNAseq data (S1 Fig.).

### Association results of 12T ins/del (*ATP1A1*) polymorphism with essential hypertension

For our genetic analysis we utilize a case control paradigm focusing on the extreme of the population as contrasting samples, i.e.: hypertensives with BP > 160/95 mmHg and normotensives subjects with BP < 138/85 mmHg. Since essential hypertension is a late-onset disease, to exclude erroneous control subjects with late-onset hypertension, normotensive controls were limited to those older than 60 years of age. To ascertain phenotype accuracy, we used stringent, clinically pertinent criteria to distinguish hypertensive cases and normotensive controls, thus ascertaining a robust case-control cohort. Phenotypic characteristics of the Sardinian sample are shown in Table 1. Stratification by gender did not significantly change the mean BP values when comparing hypertensives versus normotensives (Table 1).

**Table 1:** Characteristics of the study population.

**Variable**	**NT^a^ (total)**	**HT^b^ (total)**	**Male NT**	**Female NT**	**Male HT**	**Female HT**
n	279	433	131	148	237	196
Age, y^c^	65.4 ± 10.6	51.0 ± 10.2	66.1 ± 8.9	64.8 ± 11.9	51.8 ± 10.6	50.0 ± 9.6
BMI^d^, Kg^e^/m^2^	26.2 ± 3.9	27.7 ± 4.0	26.3 ± 3.0	26.2 ± 4.6	28.0 ± 3.8	27.4 ± 4.3
SBP^f^, mmHg^g^	127.6 ± 11.3	174.4 ± 14.7	127.9 ± 10.7	127.4 ± 11.9	173.2 ± 14.6	175.9 ± 14.8
DBP^h^, mmHg	77.6 ± 7.2	110.5 ± 9.9	77.2 ± 6.8	78.0 ± 7.4	111.9 ± 10.4	108.8 ± 9.0

^a^, Normotensives

^b^, hypertensives; total, male + female subjects

^c^, years

^d^, body mass index

^e^, kilogram per meter squared

^f^, systolic blood pressure

^g^, millimeters mercury

^h^, diastolic blood pressure.

After identification, we next tested whether the 12T ins/del polymorphism per se is associated with susceptibility to hypertension in our case-control cohort. As shown in Table 2, the 12T-ins (minor) allele is protective for hypertension susceptibility in our Sardinian cohort and exhibits sex-specificity with significantly increased frequencies of *ATP1A1* 12T-ins alleles only in males (*P* = 0.035, OR = 0.50, Table 2). To further corroborate these association results we analyzed the *ATP1A1* 12T-ins/del polymorphism based on blood pressure as a quantitative trait. Consistent with the results obtained in the case-control analysis, male subjects homozygous and heterozygous for the *ATP1A1* 12T-ins allele had lower blood pressure (−12.1 mmHg for SBP, *P* = 0.02; −6.6 mmHg for DBP, *P* = 0.046, Table 3) than homozygous carriers for the *ATP1A1* 12T-del allele. Allele-specific differential BP levels suggest that the 12T-ins/del polymorphism is most likely a functional variant which contribute to differential susceptibility to essential hypertension in males in the Sardinian cohort studied.

**Table 2:** *ATP1A1* single variant association results.

Polymorphism	Alleles	Protective allele	Controls		Cases		OR (95% c.i.) A/A vs. Aa + aa	*P* value
			Freq^a^	AA/Aa/aa^b^	Freq^a^	AA/Aa/aa^b^		
Male + Female cohort								
12T-ins/del	38T/26T	38T	0.109	209/40/8	0.071	369/46/7	0.63 (0.41–0.96)	0.035
Female cohort								
12T-ins/del	38T/26T	38T	0.109	114/18/6	0.078	165/24/3	0.78 (0.43–1.42)	0.442
Male cohort								
12T-ins/del	38T/26T	38T	0.109	95/22/2	0.065	204/22/4	0.50 (0.28–0.93)	0.035

Genotype counts for cases and controls are shown.

^a^, Minor allele frequency (38T)

^b^, For 12T-ins/del, the genotype counts are for 12T-del12T-del/12T-del12T-ins/12T-ins12T-ins

OR, odds ratio

c.i., confidence interval

*P*, Two sided Fisher’s exact *P*. *ATP1A1*, α1 Na,K-ATPase.

**Table 3:** Analysis of *ATP1A1* (12T-ins/del) variants based on blood pressure as a quantitative trait.

Genotypes	n^a^	Mean SBP^b^ ± s.e.m.	Δ SBP	*P*	Mean DBP^c^ ± s.e.m.	Δ DBP	*P*
Male ± Female cohort							
12T-del/del	577	158.8 ± 1.5			99.9 ± 1.0		
[12T-ins/del ± 12T-ins/ins]	101	151.2 ± 3.2	7.3	0.056	95.6 ± 2.3	4.3	0.078
Female cohort							
12T-del/del	279	156.7 ± 2.2			95.9 ± 1.4		
[12T-ins/del ± 12T-ins/ins]	51	154.2 ± 4.6	2.5	0.707	94.3 ± 2.6	1.6	0.798
Male cohort							
12T-del/del	298	160.2 ± 2.0			103.5 ± 1.4		
[12T-ins/del ± 12T-ins/ins]	50	148.1 ± 4.5	12.1	0.020	96.9 ± 3.7	6.6	0.046

Blood pressures were adjusted for age, body mass index and case/control status.

^a^ Number of individuals

^b^ Systolic blood pressure in mmHg

^c^ Diastolic blood pressure in mmHg

s.e.m., standard error of the mean

Δ SBP, difference in systolic blood pressure

Δ DBP, difference in diastolic blood pressure

*P*, Mann-Whitney Rank Sum Test *P* values.

### Transcriptional activity of *ATP1A1* promoter regions containing the 12T-ins/del alleles

Since the polymorphism is located close to a TATAAA-box in the promoter region, we next tested the differential effects of 12T-insertion versus 12T-deletion on transcriptional activity of the *ATP1A1* promoter using promoter-reporter minigene constructs (Fig. 1C). We used three cell lines: two of renal origin (HEK293, human embryonic kidney cell line; Cos1, monkey kidney cell line) and a human mammary tumor cell line (MDA-MB-468). As shown in Fig. 1D, the *ATP1A1* p12T-ins minigene construct (testing the 12T-insertion allele conferring protection to high blood pressure) exhibited significantly lower transcriptional activity than the p12T-del minigene construct (12T-deletion allele associated with high blood pressure) in the three cell lines tested (in Cos1, *P* < 0.05; in HEK293 and MDA-MB-468, *P* < 0.01). These observations show that the 12T-ins/del is a functional variant that modulates *ATP1A1* transcription, hence impacting levels of ATP1A1 expression with the 12T-insertion expected to decrease α1Na,K-ATPase levels, and the 12T-deletion expected to increase it. One would therefore predict that *in vivo*, a decrease in ATP1A1 expression would lead to a decrease in blood pressure in male subjects and vice versa, an increase in ATP1A1 levels would result in an increment in blood pressure, given that the α1Na,K-ATPase is involved in renal Na-reabsorption, and hence sodium levels.

### Effect of ATP1A1 haploinsufficiency on blood pressure

**Figure 2:**
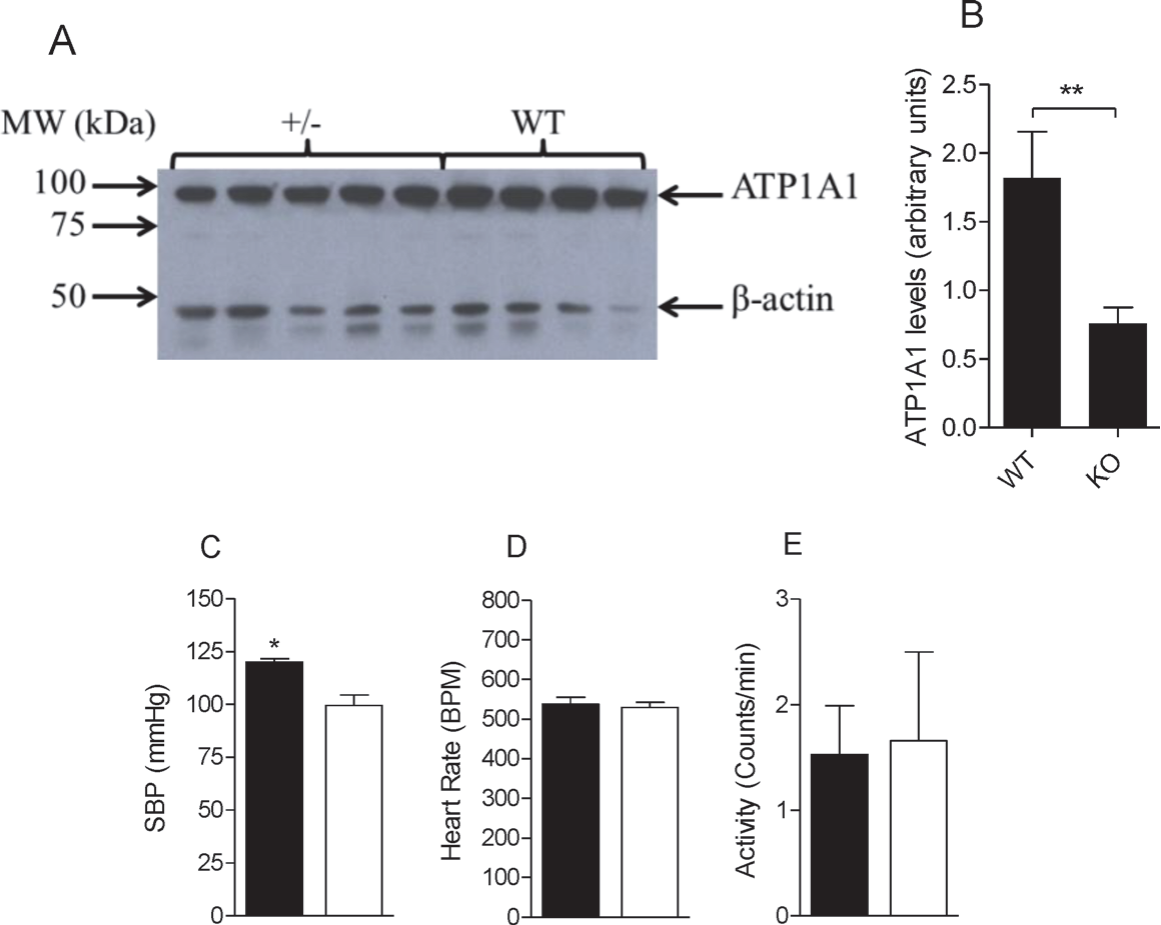
Analysis of ATP1A1 protein expression, blood pressure, heart rate and activity in heterozygous *ATP1A1^+/−^* and wild-type male mice. (A) Western blot analysis of *ATP1A1^+/−^* and wild type mouse whole kidney extracts (30 μg) reacted with anti-mouse ATP1A1 and anti-mouse βActin polypeptides. (B) Densitometry analysis of samples shown in (A) detecting 58% decrease ATP1A1 levels in *ATP1A1^+/−^* kidneys. (C) Mean systolic blood pressure ± sem (SBP; mmHg). (D) Mean heart rate ± sem (beats/min; BPM). (E) Mean activity ± sem (Counts/min) in *ATP1A1^+/−^* (solid bars, n = 4) and wild-type (open bars, n = 5) male mice. * *P* < 0.03, ** *P* = 0.015 (two-tailed student *t*-test).

To assess if changes in ATP1A1 levels could modulate blood pressure *in* vivo, in a manner that is consistent with the 12T-ins/del functional variants characterized in *in vitro* transcriptional assays we measured blood pressure by radiotelemetry in four heterozygous *ATP1A1* knockout mice (*ATP1A1^+/−^*) and five wild type male mice expressing differential ATP1A1 protein levels. Because of differential blood pressure levels in non-inbred mice, we first inbred the *ATP1A1* knockout mouse line to greater than 99.5% C57BL/6 background. Since the association is male-specific, we then studied 3-month old male heterozygous *ATP1A1^+/−^* knockout mice compared to age-matched littermate wild-type *ATP1A1^+/+^* male mice on regular mouse chow. Analysis of ATP1A1 protein levels by western blot analysis revealed that heterozygous *ATP1A1^+/−^* knockout male mice expressed 58% less ATP1A1 protein levels in kidney (Fig. 2A-2B) compared to wild type mice, as expected from haploinsufficiency. These data are concordant with decreased ATP1A1 levels in the heart of non-inbred *ATP1A1^+/−^* heterozygous knockout mice [20].

To study the effects on blood pressure, we obtained radiotelemetric blood pressures in 3-month old, inbred male heterozygous *ATP1A1^+/−^* knockout and wild-type littermate mice during a 24-hour no-disturbance period. As predicted, the heterozygous *ATP1A1^+/−^* mice exhibited lower systolic blood pressures (mean ± sem 107.63 ± 4.8 mmHg) compared to male wild-type controls (124.42 ± 3.8 mmHg; *P* < 0.03) (Fig. 2C). Notably, we detected equivalent measures of heart rate (Fig. 2D) and activity (Fig. 2E). These *in vivo* mouse model studies show that the genetic decrease in ATP1A1 expression resulted in lower blood pressure set-points, consistent with predictions from ATP1A1’s known role in Na-reabsorption in the kidney [5,6], and the causal role of increased sodium levels on endothelial stiffness [17,18].

## Discussion

Identification of genes underlying susceptibility to essential hypertension in humans has been a challenge due to its complex multifactorial etiology involving multiple genetic and environmental factors, and compounded by gene-gene and gene-environment interactions, developmental programing and gender-specific modulation. These characteristics make it difficult to elucidate causative genes with a single approach, thus requiring multiple lines of evidence involving genetic, biochemical and biological experimentation to establish causation. *A* priori, in order to demonstrate a hypertension susceptibility gene one should provide at the minimum evidence for: 1) genetic association of the gene in question with essential hypertension in a human population, 2) elucidation and genetic association of specific functional variants that might contribute to susceptibility or resistance to high blood pressure and 3) demonstration in different biological experimental systems that the effect of specific functional variants on blood pressure are consistent with the genetic association results, and concordant with known physiological functions [2,3]. In compliance with all three *a priori* requirements for the definition of a susceptibility gene in a complex trait, here we present experimental data encompassing genetic, biochemical and an *in vivo* biological system that collectively support the hypothesis that the *ATP1A1* gene is a hypertension susceptibility gene in males in a Sardinian cohort. We found a 12T-insertion (ins) polymorphism involving a poly-T sequence (38T *vs* 26T) within the *ATP1A1* 5’-regulatory region that is associated with decreased susceptibility to essential hypertension in a male Sardinian cohort, and causes decreased *ATP1A1* transcriptional activity, hence decreased ATP1A1 expression, in *in vitro* reporter-assays compared with the *ATP1A1* promoter containing the 12T-deletion. Based on α1Na,K-ATPase key role in renal Na-reabsorption [5,6] and the role of sodium in hypertension [5] and endothelial stiffness [17,18], the expected functional consequences of lower blood pressure due to lower ATP1A1 expression was demonstrated by lower blood pressure in *ATP1A1^+/−^* male mice haploinsufficient for ATP1A1 with 58% reduction in ATP1A1 protein in kidneys.

Importantly, these findings are concordant with cumulative evidence obtained in animal models of polygenic hypertension linking *ATP1A1* to salt-sensitive hypertension [8–12] albeit with different molecular basis, and human studies showing association of the *ATP1A1* locus with hypertension [14–16]. Additionally, the sex-specific effects of the *ATP1A1* promoter variants on hypertension susceptibility are also consistent with our studies in the Dahl rat model of salt-sensitive hypertension showing stronger linkage of the *ATP1A1* locus with salt-sensitive hypertension in the F2 male population [12].

Moreover, since the α1Na,K-ATPase isoform, encoded by the ATP1A1 gene, is the sole active sodium (Na) transporter in the renal basolateral epithelia throughout the nephron [4–6], any changes in ATP1A1 levels and/or activity will impact renal Na reabsorption, hence Na homeostasis and blood pressure [4–6]. The molecular phenotype of the *ATP1A1* 12T-insertion variant (protective allele) is consistent with its association with normotension (lower blood pressure) since the *ATP1A1* promoter harboring the 12T-insertion polymorphism exhibited lower transcriptional activity which *a priori* results in lower renal ATP1A1 RNA and hence, lower ATP1A1 protein levels which predicts lower blood pressure. Importantly, this *a priori* expectation was confirmed in in vivo model studies of highly inbred heterozygous *ATP1A1^+/−^* male mice with 58% reduced levels of renal α1 Na,K-ATPase and significantly lower blood pressure compared to age-matched male wild type control mice. Additionally, since increasing sodium levels within known ‘normal range from 139 mM to 145 mM sodium is associated with increasing endothelial stiffness in vitro (3–25% increase [17,18,21], collective observations raise the hypothesis lower ATP1A1 transcription and expression could also decrease endothelial stiffness via maintaining lower sodium levels given the same sodium-intake and/or via dampening the crosstalk between endothelial ENac (epithelial Na-channels) and α1Na,K-ATPase [22].

From a molecular genetic perspective, increasing evidence of insertion-deletion polymorphisms in gene promoter regions affecting susceptibility to complex disorders suggest that this type of variants are more common than originally considered. A six-nucleotide insertion-deletion polymorphism in the *CASP8* promoter has been associated with susceptibility to multiple cancers with the deletion variant having reduced caspase-8 activity and conferring decreased risk of cancer [23]. Similarly, a five-nucleotide insertion-deletion polymorphism in the *IRF5* promoter was found to be associated with inflammatory bowel disease with the insertion allele enhancing the risk of inflammatory bowel disease [24]. A functional 44 bp ins/del polymorphism in the promoter region of *5-HTTLPR* (serotonin transporter) affecting 5-HTTLPR expression has been associated with major depressive disorder [25]. More recently, a functional ATTG ins/del polymorphism in the *NFKB1* 5’-regulatory region modulating NFKB1 protein levels has been related to the risk of coronary heart disease [26] and risk of multiple cancers [27–29]. Interestingly, an ins/del polymorphism within the p21 (Waf1/Cip1) promoter region involving a poly-T sequence (11T/9T) has been associated with gastric cancer [30], however the functionality of this polymorphism has not been determined. Thus, our finding of an association of a 12T-ins/del polymorphism involving a poly-T sequence (38T/26T) with essential hypertension is not unprecedented, and is concordant with the inherent potential of a functional variant in a gene’s regulatory promoter region to provide a molecular mechanism for gene-environment interactions that result in a disease-phenotype only when both variant and environmental factor are present.

Although, the precise molecular mechanism as to how the 12T-ins/del polymorphism affects the transcription initiation complex remains to be elucidated, susceptibility-polymorphisms in gene regulatory promoter regions provide a putative mechanism for gene-environmental factor interactions through the differential modulation of transcription specific to the susceptibility-variant and environmental factor interaction. This notion would imply that replication in other cohorts could be dependent on similar gene-environment interactions including developmental programming, and that phenotyping of hypertension cohorts need to incorporate environmental factor exposure and developmental programming. In retrospect, the Sardinian island cohort provides relative homogeneity in terms of genetic background and environmental factors.

Genome-wide association studies involving large populations have failed to detect significant association between ATP1A1 and high blood pressure [31]. A number of reasons may account for this negative finding, including intrinsic genetic heterogeneity of human populations, differential accuracy and/or modality in trait measurements (blood pressure), differential exclusion criteria for affected individuals, exclusion of putative sex-specific effects on the phenotype in the analytical paradigm, and gestational risk factors. For our genetic analysis we utilized a case control paradigm focusing on the extreme of the population as contrasting samples (hypertensives with BP > 160/95 mmHg versus normotensives subjects with BP < 138/85 mmHg). Moreover, since essential hypertension is a late-onset disease, we limited normotensive controls to those older than 60 years of age to exclude erroneous control subjects with late-onset hypertension. This strategy has been predicted to be a robust approach for gene-association discovery [32]. Finally, we selected a northern Sardinian cohort for our studies as it is a relatively isolated genetic population [33,34], thus reducing putative confounders from genetic background heterogeneity and environmental factor variability. Our approach has been successful in detecting strong associations not only for ATP1A1 but also for DEspR (dual endothelin1/ vascular endothelial growth factor-signal peptide receptor) [35], NLRP6/AVR (NLR family, pyrin domain containing 6/angiotensin-vasopressin receptor) and ADM (adrenomedullin) [36] in the same Sardinian sample. It will be important to consider these factors in testing not only the ATP1A1 12T-ins/del polymorphism but also other functional gene variants for possible association with essential hypertension in other populations.

In summary, we have identified a 12T-insertion/deletion variant in the *ATP1A1* promoter region that is associated with decreased/increased susceptibility to essential hypertension in males in a northern Sardinian population. The 12T-insertion variant exhibited decreased transcriptional activity that could result in reduced expression of renal and endothelial α1 Na,K-ATPase and eventually lower blood pressure among subjects harboring this variant. Although replication in other human populations remains to be performed, our findings support the hypothesis that the *ATP1A1* gene is a susceptibility locus for essential hypertension in the setting of gene-environment interactions and phenotype-genotype context embodied in the Sardinian cohort studied.

## Materials and Methods

### Ethics Statement

The Sardinian cohort study was performed in strict accordance with the principles expressed in the Declaration of Helsinki. The protocol was approved by the local ethics committee of Local Health Unit-University of Sassari Medical School. Written informed consent was obtained and all clinical investigation was conducted according to the principles expressed in the Declaration of Helsinki. The animal studies were performed in strict accordance with the recommendations in the Guide for the Care and Use of Laboratory Animals of the National Institutes of Health. The protocol was approved by the Institutional Animal Care and use Committee on the Ethics of Animal Experiments of Boston University School of Medicine (Permit Number: AN-14965). All surgery was performed under appropriate anesthesia, and every effort was made to minimize suffering.

### Study population

The study cohort has been previously described [14,16,35,36]. It consists of 712 subjects, 433 hypertensives and 279 normotensives, all enrolled at the Hypertension and Cardiovascular Prevention Center of the University of Sassari Medical School, Sassari, Sardinia, Italy. Studies were approved by the local ethics committee of Local Health Unit-University of Sassari Medical School. All subjects were white, unrelated, born in different domains of North Sardinia previously ascertained to have a high degree of genetic homogeneity [33,34], ascertained to be Sardinian for at least 6 generations, and resided in Sardinia. Hypertensive patients with BP > 160/95 mmHg (n = 433), no secondary hypertension etiology and absence of major comorbid conditions were considered in the study. Older patients were included only if they were diagnosed as hypertensive well before 55 years of age. BP measurements were obtained with patients not taking any medications. Family history of hypertension was investigated, and a complete pedigree was defined. To exclude erroneous control subjects with late-onset hypertension, normotensive controls (n = 279) were limited to those older than 60 years of age who had not been previously diagnosed or treated as hypertensive, had no family history of hypertension and cardiovascular or cerebrovascular disease, and had BP values < 138/85 on at least 4 occasions.

### Cloning and sequencing of *ATP1A1* 5’-regulatory regions

We cloned and sequenced a 4551 bp DNA fragment using the following primers: forward primer: 5′-AGA-TCA-TGA-GGC-TGA-GTG-AG-3′ and reverse primer: 5′-TTC-CAT-TTT-GGC-GAT-GGT-G-3′) encompassing chromosome-1 coordinates 116363538–116368089 (Homo sapiens chromosome 1 GRCh38 Primary Assembly) of the 5’-regulatory region of *ATP1A1* (Fig. 1) from six patients, three carrying haplotypes associated with hypertension and three carrying haplotypes associated with normotension respectively. Each fragment was subcloned into the PT-vector system (Clontech, Palo Alto, CA) and then sequenced in its entirety in both directions.

### Transcriptional activity of *ATP1A1* promoter regions

We measured transcriptional activity as described [35] using the SEAP Reporter System (Clontech Lab,Inc.) which measures secreted alkaline phosphatase (SEAP) produced by the transfected minigene construct. We cloned the ATP1A1 promoter regions onto pSEAP2 vector generating two types of constructs: one carrying the 12T-insertion polymorphism (p12Tins, Fig. 1C), and one containing the 12T-deletion polymorphism (p12Tdel, Fig. 1C). We measured transcriptional activity in three cell lines, two of renal origin (HEK293, human embryonic kidney cell line; Cos1, monkey kidney cell line) and one human mammary tumor cell line (MDA-MB-468, ATCC) since ATP1A1 is expressed in all tissues and prominently in kidney [4–6].

Transfections were performed as described [35]. Cells were co-transfected with 3.0 μg of 5’-regulatory region-SEAP constructs plus 1.5 μg of pSV2-β-Galactosidase (for internal control) using the DOTAP liposomal transfection reagent (Roche Molecular Biochemicals). After 72 hours, cell culture supernatants were collected and assayed for SEAP activity following manufacturer’s specifications. Cell protein extracts were utilized for determination of β-Galactosidase activity (Promega) to normalize SEAP activity. Each construct was tested in six replicates in the presence of standard tissue culture conditions containing 156 mM Na+ in the media, which is expected to simulate high sodium levels that increase endothelial stiffness since levels above 139 mM sodium increases endothelial stiffness [21].

### Blood pressure measurements in *ATP1A1^+/−^* and wild type mice

We used B6;129S5-*Atp1a1^Gt(neo)311Lex^* mice (stock number: 011687-UCD) procured from the Mutant Mouse Regional Resource Center (MMRRC) supported by NCRR-NIH. This *ATP1A1* knockout line was developed by targeted retroviral insertion which occurred between exons 1 and 2 of the mouse *ATP1A1* gene. Genetic data indicate that this retroviral insertion resulted in lethality of the homozygous mutants. The line is maintained as a heterozygous line and available with a mixed genetic background: 129/SvEvBrd × C57BL/6 hybrid. We inbred this line onto C57BL/6 genetic background by speed-congenic backcross breeding as described [37]. Briefly, we selected 20 males from a BC1 (backcross 1; B6;129S5-*Atp1a1^Gt(neo)311Lex^* × C57BL/6) generation that were genotyped with 142 SNP markers (custom performed by The Jackson Laboratory/JAX Genome Scan service) informative for the 129/SvEvBrd × C57BL/6 cross and selected the best male (carrying the least 129/SvEvBrd genetic background) as breeder for the production of a BC2 generation. Backcross breeding continued for additional four generations, BC5, which provided all *ATP1A1^+/−^* mice for analyses with > 99.5% C57BL/6 genetic background. Animals utilized for BP measurements were 3 months of age. Five wild-type and four *ATP1A1^+/−^* littermate male mice were produced for the study from the same BC5 (+/+) × (+/−) intercross. Radiotelemetric blood pressures were obtained as described [35]. Surgical implantation into the carotid artery was done per manufacturer’s specifications using mouse radiotelemetric implants (PA-C10, Dataquest A.R.T. 4.2 system from DATA SCIENCES INTERNATIONAL). Briefly, we obtained blood pressure measurements taking the average over ten-seconds every 5 minutes for 24 hours with no personnel entry during the time of recording. Systolic (SBP), diastolic (DBP) and mean arterial pressures (MAP) were obtained along with heart rate and activity. Ideal conditions were ascertained: from the time of surgery. In contrast to common practice in published literature, implanted mice were housed individually in cages placed over the telemetric receiver systems in a room dedicated 24/7 for telemetric BP measurements with no other mouse cages in the room other than the study mice. Importantly, since in vivo mouse studies were done in a dedicated dual housing-measurement room, there are no confounders from having to move mice from their housing room to a BP-telemetry room for measurement.

### Western blot analysis

We analyzed 3 month-old *ATP1A1^+/−^* (n = 5 males) and wild type (n = 4 males) mice for ATP1A1 protein levels by Western blot analysis as described [38], using equal amounts of protein (30μg) from mouse whole kidney extracts. A mouse monoclonal IgG anti-ATP1A1 specific antibody (ab) (sc-21712, Santa Cruz Biotechnology; 1:500 dilution, primary antibody incubation for 16 hours at 4°C) was used to detect ATP1A1-specific polypeptide [38]. An anti-βActin specific antibody (sc-47778, Santa Cruz Biotechnology) was used as control for densitometry analysis.

### Genotyping

Genotyping was performed with a PCR-based *ATP1A1*-specific fragment (174 bp). The PCR product (forward primer: 5′-ACT-GTG-TCC-ATG-ACC-AGA-C-3′; reverse primer: 5′-TTC-AAG-ACC-AGC-CTG-AGT-G-3′) contained the 12T-ins/del polymorphism within the 5’regulatory region. Alleles (12T-ins and 12T-del) were identified and distinguished from each other by 6% sequencing polyacrylamide gel electrophoresis (Fig. 1A) as described [16]. The genotyping completeness rate was 87%.

### Statistical analysis

Single point association analysis comparing case and control subjects was done with the SNP & Variation Suite genetic analysis software (version 6.4.3, released on 03-25-2009, Golden Helix Inc., Bozeman, MT, USA). A basic allelic test (D vs d) was implemented using Fisher’s exact test as statistical method obtaining odds ratios with corresponding confidence limits. The missing genotypes were not included (imputed) in the association analysis. Analysis of blood pressure as a quantitative trait was performed using two-sided student *t*-test (SigmaPlot 11.0) comparing blood pressure levels in homozygous 12T-del/del carriers versus subjects carrying [12T-ins/del + 12T-ins/ins] genotypes.

## Supporting Information

S1 ARRIVE Checklist (DOCX)

S1 Fig Location of putative *ATP1A1* RNA initiation site. (A) Human chromosome 1 map (116363000–116405000) with *ATP1A1* location notated (*Homo sapiens* Annotation Release 106), along with Hs RNA map and RefSeq RNA map spanning *ATP1A1* gene. Potential RNA initiation site is noted at 116366000 with predicted exon 1 (5’-Untranslated region) supported by RNAseq data. (B) Human chromosome 1 map (116363000–116375000) encompassing *ATP1A1* 5’-region.
(PDF)
