# Supplementary material for: A Functional 12T-Insertion Polymorphism in the ATP1A1 Promoter Confers Decreased Susceptibility to Hypertension in a Male Sardinian Population
Source: PLoS One. 2015 Jan 23;10(1):e0116724. doi: 10.1371/journal.pone.0116724 (PMC4304799; doi:10.1371/journal.pone.0116724)

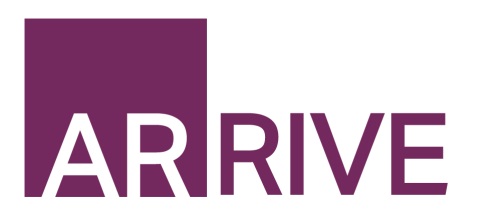


The ARRIVE Guidelines Checklist

Animal Research: Reporting In Vivo Experiments

Carol Kilkenny^1^, William J Browne^2^, Innes C Cuthill^3^, Michael Emerson^4^ and Douglas G Altman^5^

*^1^The National Centre for the Replacement, Refinement and Reduction of Animals in Research, London, UK, ^2^School of Veterinary Science, University of Bristol, Bristol, UK, ^3^School of Biological Sciences, University of Bristol, Bristol, UK, ^4^National Heart and Lung Institute, Imperial College London, UK, ^5^Centre for Statistics in Medicine, University of Oxford, Oxford, UK.*

|  | | ITEM | RECOMMENDATION | Section/ Paragraph |
| --- | --- | --- | --- | --- |
| 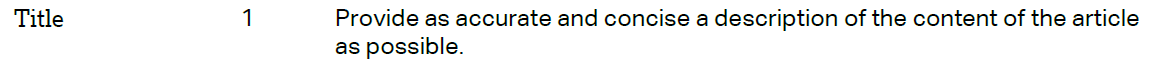 | | | Title |  |
| 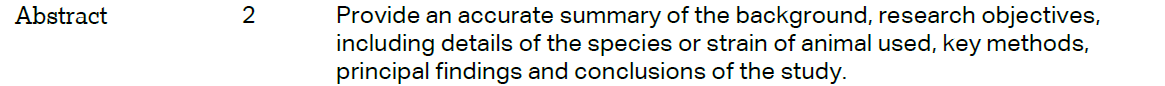 | | | Abstract |  |
| INTRODUCTION | | |  |  |
| 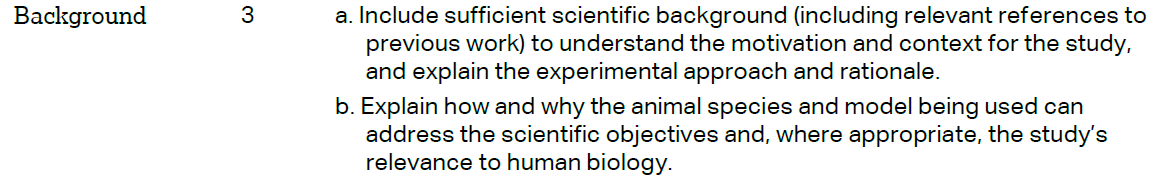 | | | Paragraphs 1-2 |  |
| 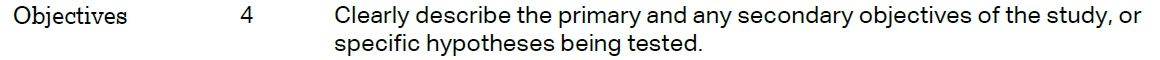 | | | Paragraph 2 |  |
| METHODS | | |  |  |
| 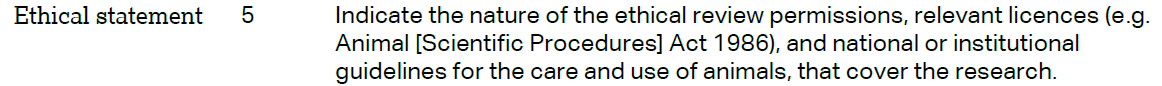 | | | Paragraph 1 |  |
| 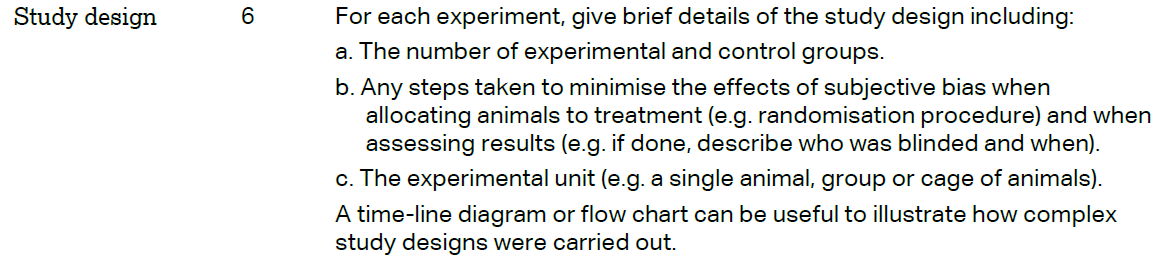 | | | Paragraph 5 |  |
| 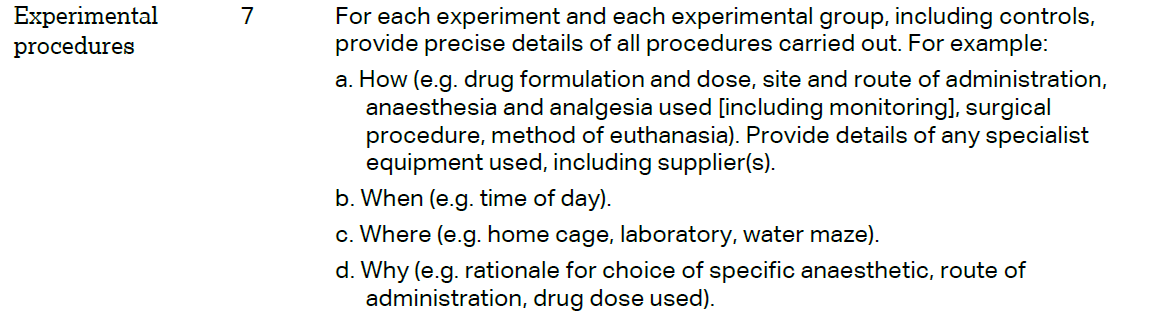 | | | Paragraph 5 |  |
| 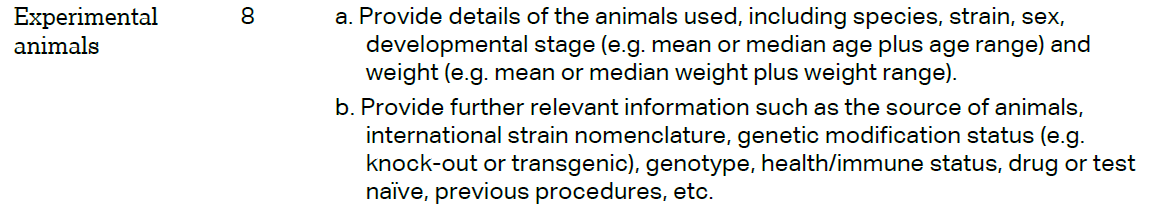 | | | Paragraph 5 |  |

The ARRIVE guidelines. Originally published in *PLoS Biology*, June 2010^1^

| 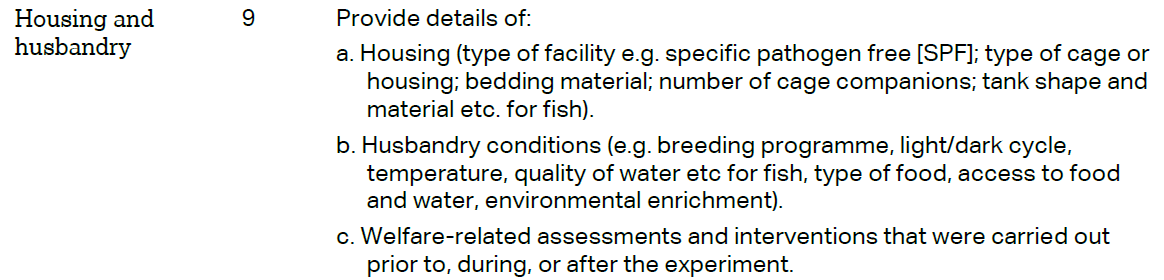 | Paragraph 5 | |
| --- | --- | --- |
| 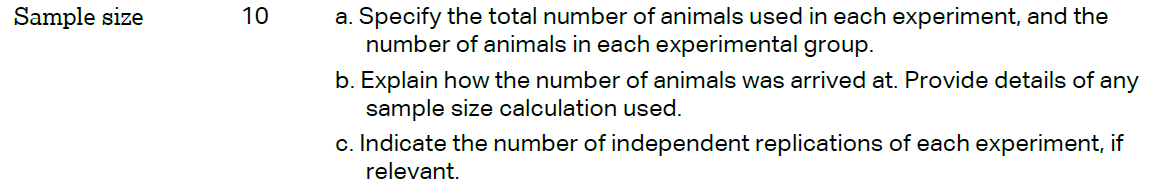 | Paragraph 5 and Figure 2 legend | |
| 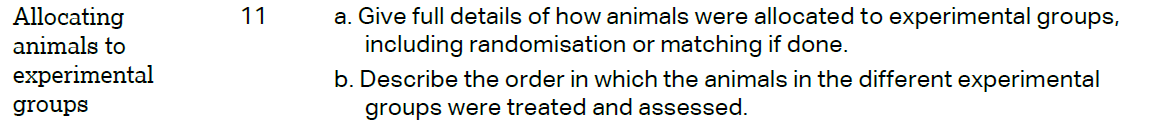 | Paragraph 5 | |
| 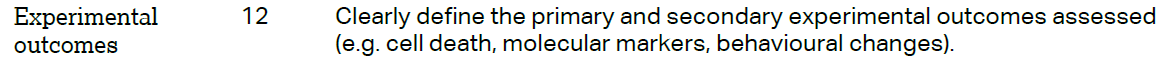 | Paragraph 5 | |
| 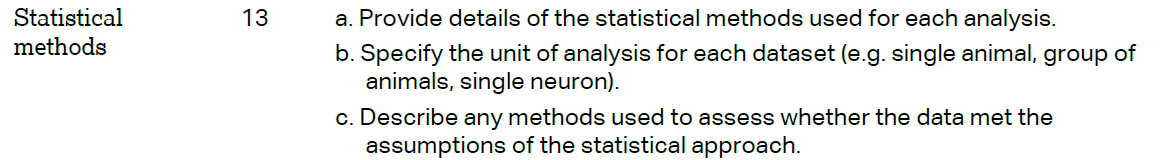 | Figure 2 legend | |
| RESULTS |  | |
| 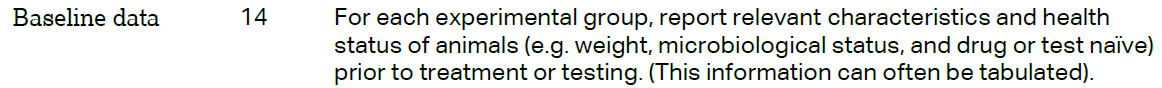 | N/A | |
| 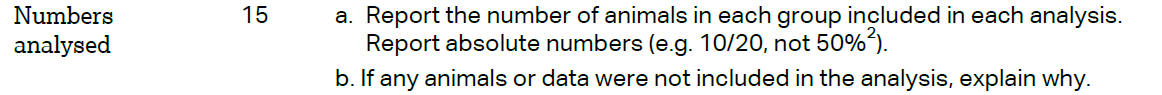 | Paragraph 6 and Figure 2 legend | |
| 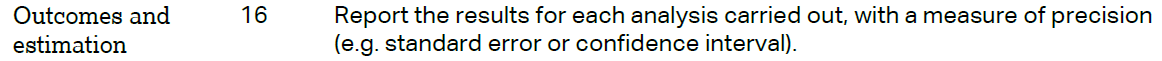 | Paragraph 7 | |
| 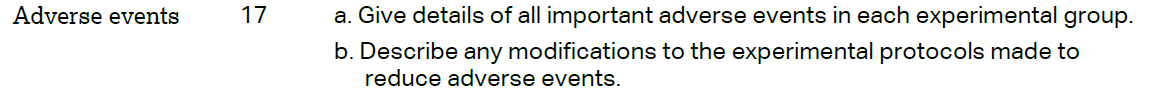 | N/A | |
| DISCUSSION |  | |
| 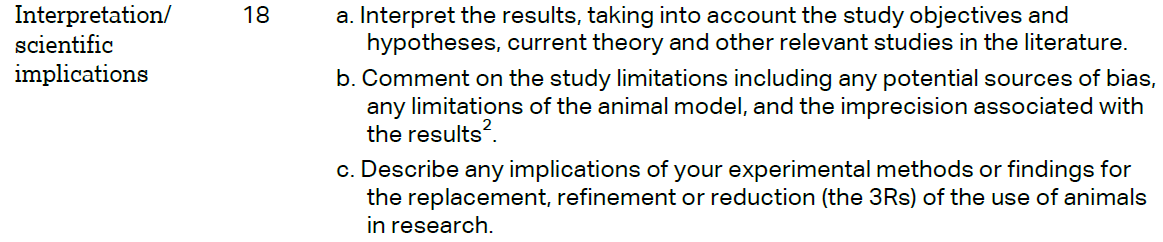 | Paragraph 1 | |
| 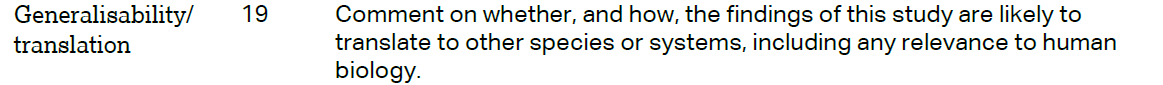 | Pargraphs 1-3 | |
| 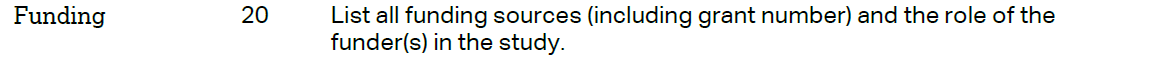 | | Provided in PLoS One submission system |


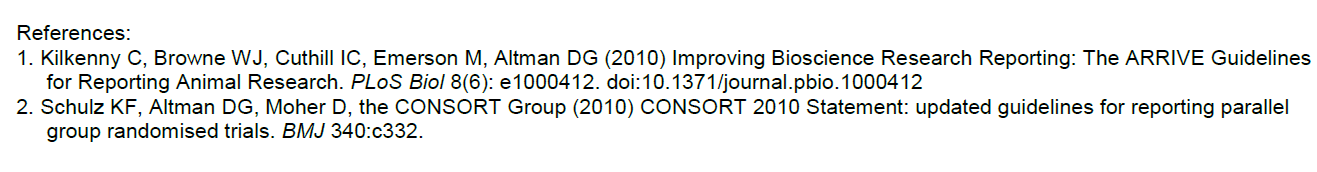

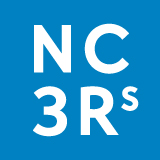

Supplement: S1 ARRIVE Checklist — (DOCX) [file pone.0116724.s001.docx]
