# Supplementary figures and images for: A Functional 12T-Insertion Polymorphism in the ATP1A1 Promoter Confers Decreased Susceptibility to Hypertension in a Male Sardinian Population
Source: PLoS One. 2015 Jan 23;10(1):e0116724. doi: 10.1371/journal.pone.0116724 (PMC4304799; doi:10.1371/journal.pone.0116724)

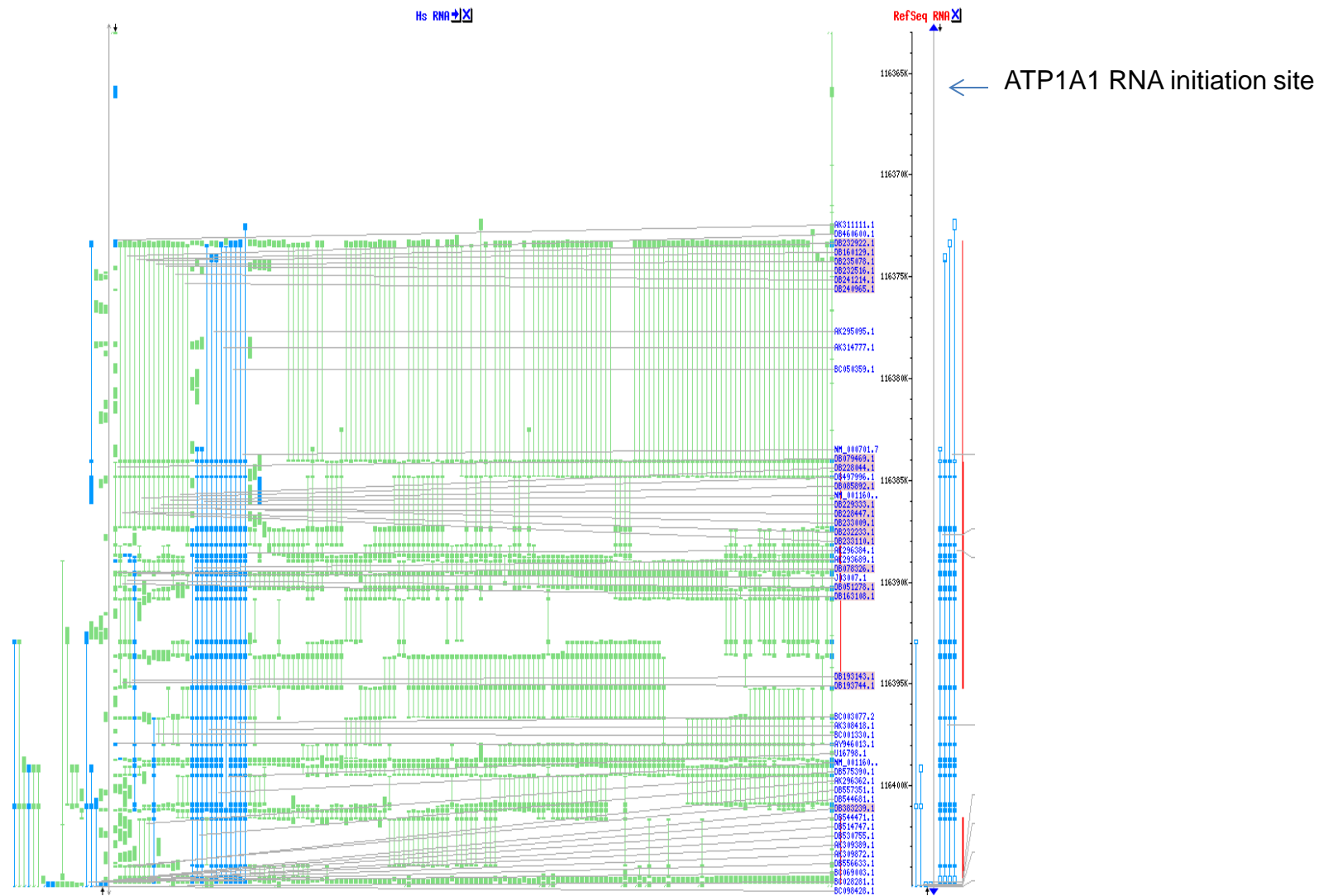

Figure S1A

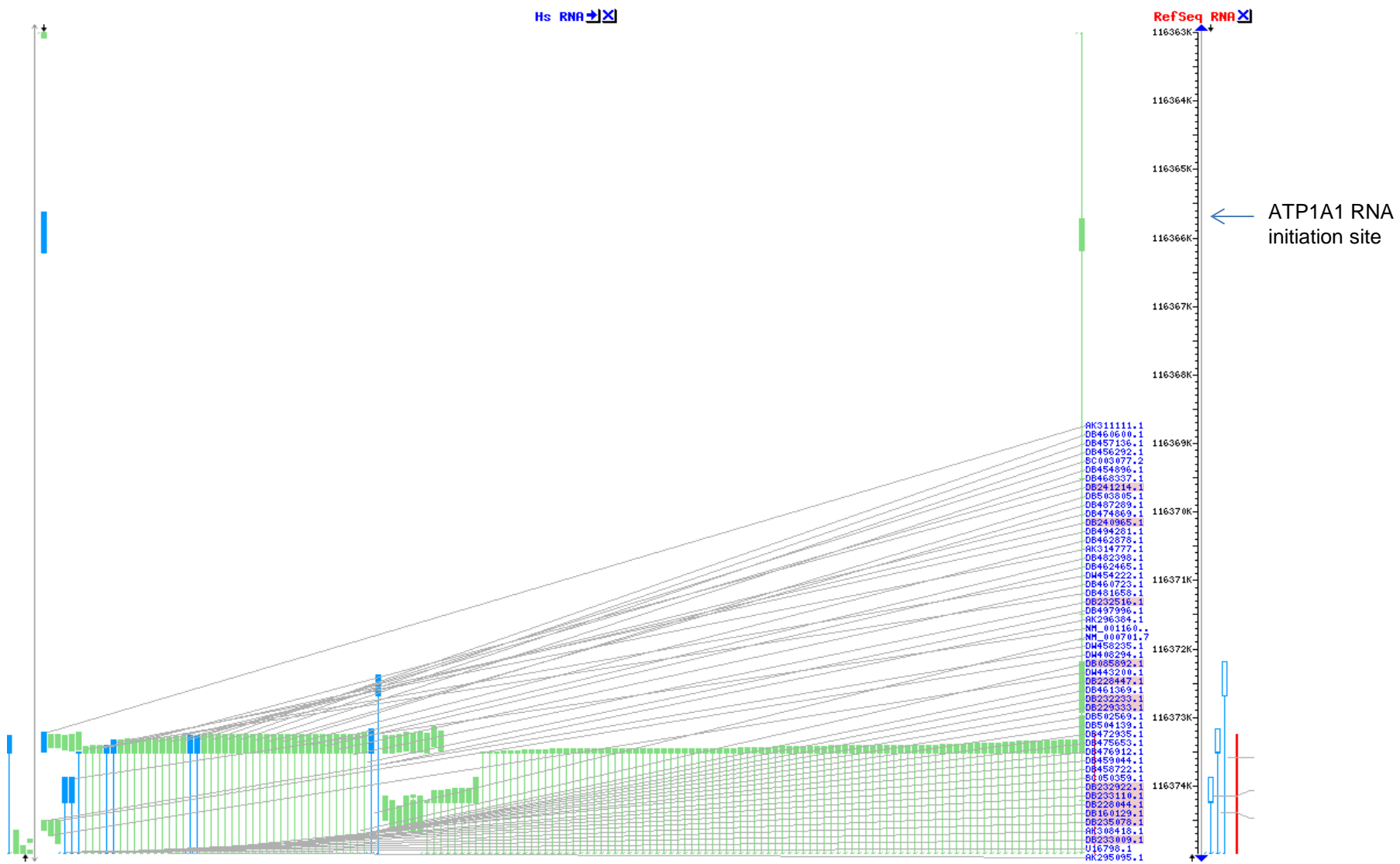

Figure S1B

Supplement: S1 Fig — (A) Human chromosome 1 map (116363000–116405000) with ATP1A1 location notated (Homo sapiens Annotation Release 106), along with Hs RNA map and RefSeq RNA map spanning ATP1A1 gene. Potential RNA initiation site is noted at 116366000 with predicted exon 1 (5’-Untranslated region) supported by RNAseq data. (B) Human chromosome 1 map (116363000–116375000) encompassing ATP1A1 5’-region. (PDF) [file pone.0116724.s002.pdf]
